# The effect of medical and operative birth interventions on child health outcomes in the first 28 days and up to 5 years of age: A linked data population‐based cohort study

**DOI:** 10.1111/birt.12348

**Published:** 2018-03-25

**Authors:** Lilian L. Peters, Charlene Thornton, Ank de Jonge, Ali Khashan, Mark Tracy, Soo Downe, Esther I. Feijen‐de Jong, Hannah G. Dahlen

**Affiliations:** ^1^ Department of Midwifery Science VU University Medical Center Amsterdam Amsterdam Public Health Research Institute Amsterdam The Netherlands; ^2^ Department of General Practice & Elderly Care Medicine University Medical Center Groningen University of Groningen Groningen The Netherlands; ^3^ AVAG Midwifery Academy Amsterdam Groningen Amsterdam/Groningen The Netherlands; ^4^ College of Nursing and Health Sciences Adelaide Flinders University Adelaide SA Australia; ^5^ School of Public Health University College Cork Cork Ireland; ^6^ The Irish Centre for Fetal and Neonatal Translational Research University College Cork (INFANT) Cork Ireland; ^7^ Westmead Newborn Intensive Care Unit Westmead Hospital University of Sydney Sydney NSW Australia; ^8^ University of Central Lancashire Preston Lancashire UK; ^9^ School of Nursing and Midwifery Sydney Western Sydney University Sydney NSW Australia; ^10^ Affiliate of the Ingham Institute Liverpool NSW Australia; ^11^ National Institute of Complementary Medicine Western Sydney University Sydney NSW Australia

**Keywords:** birth interventions, child's health, epidemiology

## Abstract

**Background:**

Spontaneous vaginal birth rates are decreasing worldwide, while cesarean delivery, instrumental births, and medical birth interventions are increasing. Emerging evidence suggests that birth interventions may have an effect on children's health. Therefore, the aim of our study was to examine the association between operative and medical birth interventions on the child's health during the first 28 days and up to 5 years of age.

**Methods:**

In New South Wales (Australia), population‐linked data sets were analyzed, including data on maternal characteristics, child characteristics, mode of birth, interventions during labor and birth, and adverse health outcomes of the children (ie, jaundice, feeding problems, hypothermia, asthma, respiratory infections, gastrointestinal disorders, other infections, metabolic disorder, and eczema) registered with the International Statistical Classification of Diseases and Related Health Problems, Tenth Revision, Australian Modification codes. Logistic regression analyses were performed for each adverse health outcome.

**Results:**

Our analyses included 491 590 women and their children; of those 38% experienced a spontaneous vaginal birth. Infants who experienced an instrumental birth after induction or augmentation had the highest risk of jaundice, adjusted odds ratio (aOR) 2.75 (95% confidence interval [CI] 2.61‐2.91) compared with spontaneous vaginal birth. Children born by cesarean delivery were particularly at statistically significantly increased risk for infections, eczema, and metabolic disorder, compared with spontaneous vaginal birth. Children born by emergency cesarean delivery showed the highest association for metabolic disorder, aOR 2.63 (95% CI 2.26‐3.07).

**Conclusion:**

Children born by spontaneous vaginal birth had fewer short‐ and longer‐term health problems, compared with those born after birth interventions.

## 1 INTRODUCTION

Worldwide cesarean delivery rates are increasing, while spontaneous vaginal birth rates are decreasing.^1^ The rate of cesarean delivery has risen steadily in Europe to 25%, in Australia to 33%, and the highest rates are reported in Latin America and the Caribbean (41%).^1^, ^2^ Instrumental birth (ie, forceps or vacuum) and medical birth interventions (ie, including the use of exogenous oxytocin for labor induction and/or augmentation) are increasing globally as well.^3^

There is emerging evidence that operative birth (ie, instrumental vaginal birth or cesarean) may have an effect on children's physical health and cognitive development in the longer term.^4^, ^5^, ^6^, ^7^ The Extended Hygiene Hypothesis (EHH) hypothesizes that infants born by cesarean delivery have different colonization of the gut flora compared with infants born by vaginal birth. This may potentially affect the neonatal immune response.^8^, ^9^ The EPIgenetic Impact of Childbirth (EPIIC) hypothesis raises concern over the effects of stress (too high and too low) caused by medical and operative birth interventions to the epigenetic regulation of gene expression in the immune system.^10^, ^11^

Studies have demonstrated that children born vaginally at term have different short‐ and longer‐term physical health outcomes than those born by cesarean, particularly when there has been no exposure.^4^, ^6^, ^7^ Epidemiological studies that analyzed population‐based registry data, reported conflicting associations between operative birth interventions, and the increased risk of several immune‐related diseases, including asthma, type 1 diabetes, obesity, and inflammatory bowel disease.^12^, ^13^, ^14^, ^15^, ^16^, ^17^, ^18^ These conflicting findings may be due to different statistical methods used, differences in study population characteristics (eg, maternal age, morbidity, smoking, and gestational age), and failure to differentiate between mode of birth and medical birth interventions.

The aim of this study was to examine the associations between operative and/or medical birth interventions on children's health outcomes in the first 28 days and up to 5 years of age, in a large population of healthy pregnant women and their children.

## 2 METHODS

The study cohort consisted of women and their children born in New South Wales (NSW), Australia, between January 1, 2000 and August 31, 2008. Children's health was followed until August 31, 2013. The NSW Centre for Health Record Linkage utilized probabilistic data linkage techniques to merge data of the following data sets: Record Linkage from the Perinatal Data Collection (PDC), Admitted Patient Data Collection, Register of Congenital Conditions, NSW Registry of Birth Deaths and Marriages, the Australian Bureau of Statistics—Socio‐Economic Indexes for Areas. Probabilistic record linkage software assigns a “linkage weight” to pairs of records.^19^, ^20^, ^21^ For example, records that match perfectly or nearly perfectly on first name, surname, date of birth, and address have a high linkage weight and records that match only on date of birth have a low‐linkage weight.^19^, ^20^, ^21^ If the linkage weight is high, it is likely that the records truly match, and if the linkage weight is low it is likely that the records are not truly a match.^19^, ^20^, ^21^ This technique has been shown to have a false‐positive rate of 0.3% of records.^19^, ^20^, ^21^ Several studies have evaluated the validity of the NSW linkage data and reported a tendency toward underreporting of maternal medical conditions during pregnancy.^22^, ^23^ However, by comparing PDC and Admitted Patient Data Collection data with women's individual medical records, it showed that conditions and procedures regarding delivery and discharge status had high specificity, indicating that false positives were uncommon.^23^, ^24^

The study was approved by the Ethics Committee of the NSW Population and Health Services Research Committee (HREC/10/CIPHS/96). The ethics privacy statement outlines that consent is waived due to the size of the data set, retrospective nature of the data, and the inherent difficulties in obtaining consent.

### 2.1 Data

Data were routinely collected from women who gave birth or had subsequent births in either a public or private hospital in NSW, Australia. Data of nulliparous and multiparous women were selected if they were low‐risk pregnant women according to the guideline of the National Institute for Health and Care Excellence on intrapartum care and in alignment with methodology previously utilized on this and other linked data sets.^20^, ^25^, ^26^ This resulted in a cohort of “healthy pregnant women” who had no preexisting or pregnancy‐related hypertension or diabetes, did not smoke or take drugs, were within the age range of 20‐35 years, and gave birth at 37‐41 weeks of gestation to a singleton baby in cephalic presentation with a birthweight of ≥2500 g. In addition, children with minor or major congenital conditions were excluded based on the International Statistical Classification of Diseases and Related Health Problems, Tenth Revision, Australian Modification codes (ICD‐10‐AM, Q0.0‐Q99.9).^25^ Minor malformations could be related to more major malformations, which could be caused by genetic factors, for example, and which may become apparent some time after birth. Moreover, women and their children were excluded from the analyses if stillbirth or death had occurred during the 5‐year follow‐up period. By making this selection, we aimed to include a population of healthy pregnant women and their healthy born children, to reduce confounding and to increase the likelihood of finding the true association between exposure (birth interventions) and outcomes (child's health short and longer term).

Women or children with missing data on either mode of birth, maternal age, parity, gestational age, or birthweight were also excluded from the analyses since these variables have a potential effect on children's health. If missing data on other variables occurred, that is, country of birth, socioeconomic status, and infant gender, these variables were indicated as system missing in the linked data file and subsequently excluded from the logistic regression models.

### 2.2 Variables

Mode of birth and birth interventions were collected from the PDC file and included: spontaneous vaginal birth, instrumental birth, elective cesarean delivery, and an emergency cesarean (either with or without medical birth interventions). Medical birth interventions included induction or augmentation of labor with oxytocin, prostaglandin, and/or artificial rupture of membranes.

The short‐term follow‐up data of infants during the first 28 days and of children up to 5 years of age included admissions to public and private hospitals located in NSW (Admitted Patient Data Collection file). The children were followed from the date of birth until their fifth birthday to identify any adverse health outcomes which occurred during this time period. The classifying diagnoses and reasons with accompanying hospital visit dates were registered with ICD‐10‐AM‐codes. Short‐term adverse health outcomes included jaundice, feeding problems, and hypothermia and often are part of the adaptation of the infant to being outside the uterus.^7^, ^27^, ^28^ The longer‐term adverse health outcomes included asthma, respiratory infections (eg, common cold, pneumonia, bronchitis), gastrointestinal disorders, other infections (eg, sepsis, streptococcus, cystitis), metabolic disorder (eg, hypoglycemia, neonatal diabetes mellitus, diabetes mellitus type 1 or 2, localized adiposity), and eczema, based on the potential effect that mode of birth has on immune‐related diseases.^5^, ^6^, ^7^, ^12^, ^13^, ^14^, ^15^, ^16^, ^17^, ^18^ An overview of all adverse health outcomes and other covariates with corresponding ICD‐10‐AM codes are presented in the Supporting Information.

Potential confounders of either women or children characteristics were selected from several data files. Women's characteristics were extracted from the PDC and NSW Registry of Birth Deaths and Marriages files and included, for example, age and country of birth. From the Socio‐Economic Indexes for Areas file, the socioeconomic status of women were collected and were based on area indices of income and education using women's postal codes and defined as low (10‐30 percentiles), medium (40‐60 percentiles) or high (≥70 percentiles). Pharmacological pain medication during labor and birth (ie, nitrous oxide, systematic opioid, local administered to perineum, pudendal, morphine, and pethidine), and anesthesia (ie, epidural, caudal, and spinal) were extracted from the PDC file.

Children's characteristics were extracted from the PDC and Admitted Patient Data Collection files and included gender, gestational age, birthweight, small‐for‐gestational age, large‐for‐gestational age, and birth trauma (appendix).

### 2.3 Data analyses

A retrospective analysis of prospectively collected linked data was performed. Baseline characteristics of the women and children were reported using descriptive statistics. Statistical differences in baseline characteristics in women and children in the different mode of birth and birth interventions groupings (ie, spontaneous vaginal birth, vaginal birth with induction or augmentation, instrumental birth, instrumental birth with induction or augmentation, elective cesarean delivery, emergency cesarean, and an emergency cesarean delivery after induction) were calculated with chi‐square tests.

Univariate and multivariate logistic regression analyses were performed to examine the association between the exposure variable and each child outcome. Spontaneous vaginal birth without induction or augmentation of labor was used as the reference group. The logistic regression models were adjusted for maternal characteristics (maternal age, country of birth, socioeconomic status, parity), birth characteristics (pain medication during birth), and child characteristics (gender, gestational age, birthweight, small‐for‐gestational age, large‐for‐gestational age, birth trauma). Crude and adjusted odds ratios (OR) with corresponding 95% confidence intervals (CI) were reported. For all analyses, a *P*‐value of .01 was defined as significant and all statistical analyses were performed with SPSS Statistics 23.0 (SPSS Inc., Chicago, IL, USA).

## 3 RESULTS

The total population linked data set contained the antenatal, birth, and postnatal details of 669 880 women and 1 097 762 births which occurred in public or private hospitals during the study period 2000‐2008 with a follow‐up until age 5 years. A total 548 815 births (50%) were excluded due to medical or obstetric risk factors (eg, maternal morbidity, preterm birth) or substance abuse in pregnancy (eg, smoking or drug abuse). We excluded 54 254 (4.9%) children with congenital abnormalities. After applying all other exclusion criteria, mortality was recorded for 1638 (0.1%) children during the study period. There were 653 stillbirths, 353 cases of neonatal mortality, and 632 of childhood mortality. The risk of neonatal mortality was similar across the different mode of births. Finally, 1465 cases were excluded due to missing data on either maternal characteristics (ie, age, mode of birth, parity) or child characteristics (ie, gestational age, birthweight). The final study cohort consisted of 491 590 healthy pregnant women and their children.

The majority of the women were Australian born (69%) and had a mean age of 29 (SD 4) years. Fifty‐five percent of the women were nulliparous. About 38% had a spontaneous vaginal birth, 28% had a vaginal birth with induction or augmentation, 4% had an instrumental birth without induction or augmentation, and 8% had an instrumental birth with induction or augmentation. Eleven percent of these women had an elective cesarean delivery, 4% had an emergency cesarean, and 7% had an emergency cesarean delivery after induction or augmentation of labor (Table 1). Overall, 43% of the women included were induced before labor, or received augmentation during labor. The majority (88%) of women received pain medication and infant's birth trauma was experienced in 3% of the births. Maternal (eg, socioeconomic status), birth (eg, pain medication), and child characteristics (eg, gestational age) showed statistically significant differences across seven modes of the birth group (*P* ≤ .001, Table 1). Missing values ranged from 0.04% (ie, infant gender) to 0.3% (ie, socioeconomic status) in the final linked data set.

**Table 1:** Maternal, mode of birth, and child characteristics by type of birth interventions, New South Wales, Australia, 2000‐2008

	Total population	Spontaneous vaginal birth	Vaginal birth with induction or augmentation^a^	Instrumental birth	Instrumental birth with induction or augmentation^a^	Cesarean elective	Cesarean emergency	Cesarean emergency after induction or augmentation	Statistical differences among groups that differed on mode of birth
	N* *=* *491 590 100% N (%)	n* *=* *185 883 37.8% n (%)	n* *=* *136 651 27.8% n (%)	n* *=* *19 865 4.0% n (%)	n* *=* *41 631 8.5% n (%)	n* *=* *55 499 11.3% n (%)	n* *=* *17 216 3.5% n (%)	n* *=* *34 845 7.1% n (%)	*P*‐value
Maternal characteristics									
Maternal age (y)									
20‐24	82 638 (16.8)	360 51 (19.4)	25 482 (18.6)	2901 (14.6)	6063 (14.6)	4612 (8.3)	2175 (12.6)	5354 (15.4)	≤.001
25‐29	185 308 (37.7)	71 439 (38.4)	53 382 (38.3)	7749 (39.0)	16 514 (39.7)	17 391 (31.3)	6175 (35.9)	13 658 (39.2)	
30‐35	223 644 (45.5)	78 393 (42.2)	58 787 (43.0)	9215 (46.4)	19 054 (45.8)	33 496 (60.4)	8866 (51.5)	15 833 (45.4)	
Country of birth									
Australia	339 072 (69.0)	122 577 (65.9)	99 432 (72.8)	13 096 (65.9)	27 620 (66.3)	41 191 (74.2)	11 653 (67.7)	23 503 (67.5)	≤.001
Not Australia	151 335 (30.8)	62 915 (33.8)	36 926 (27.0)	6730 (33.9)	13 884 (33.4)	14 124 (25.5)	5508 (32.0)	11 248 (32.3)	
Missing	1183 (0.2)	391 (0.2)	293 (0.2)	39 (0.2)	127 (0.3)	184 (0.3)	55 (0.3)	94 (0.3)	
Socioeconomic status^b^									
Low	123 311 (25.1)	52 186 (28.1)	35 710 (26.1)	3762 (18.9)	7876 (18.9)	12 449 (22.4)	4000 (23.2)	7328 (21.0)	≤.001
Middle	169 198 (34.4)	65 892 (35.4)	47 549 (34.8)	6505 (32.7)	13 325 (32.0)	18 121 (32.7)	5741 (33.3)	12 065 (34.6)	
High	197 712 (40.2)	67 344 (36.2)	53 037 (38.8)	9535 (48.0)	20 222 (48.6)	24 816 (44.7)	7433 (43.2)	15 325 (44.0)	
Missing	1369 (0.3)	461 (0.2)	355 (0.3)	63 (0.3)	208 (0.5)	113 (0.2)	42 (0.2)	127 (0.4)	
Parity									
Nulliparous	219 951 (44.7)	63 906 (34.4)	56 547 (41.4)	15 163 (76.3)	34 574 (83.0)	11 560 (20.8)	8788 (51.0)	29 413 (84.4)	≤.001
Multiparous	271 639 (55.3)	121 977 (65.6)	80 104 (58.6)	4702 (23.7)	7057 (17.0)	43 939 (79.2)	8428 (49.0)	5432 (15.6)	
Mode of birth characteristics									
Pain medication									
None	57 214 (11.6)	42 889 (23.1)	13 602 (10.0)	418 (2.1)	305 (0.7)	–	–	–	≤.001
Pharmacological pain medication^a^	231 106 (47.0)	12 6011 (67.8)	82 384 (60.3)	10 080 (50.7)	12 631 (30.3)	–	–	–	
Epidural, caudal, spinal or general anesthesia	200 942 (40.9)	15 427 (8.3)	40 017 (29.3)	9326 (46.9)	28 666 (68.9)	55 461 (99.9)	17 208 (100)	34 837 (100)	
Missing	2328 (0.5)	1556 (0.8)	648 (0.5)	41 (0.2)	29 (0.1)	38 (0.1)	8 (0)	8 (0)	
Child characteristics									
Gender									
Female	242 168 (49.3)	94 616 (50.9)	69 096 (50.6)	8993 (45.3)	19 367 (46.5)	26 988 (48.6)	7650 (44.4)	14 458 (44.4)	≤.001
Male	249 242 (50.7)	91 237 (49.1)	67 517 (49.4)	10 864 (54.7)	22 246 (53.4)	28 449 (51.3)	9554 (55.5)	19 375 (55.6)	
Missing	180 (0)	30 (0)	38 (0)	8 (0)	18 (0)	62 (0)	12 (0)	12 (0)	
Gestational age									
37‐37 + 6	21 800 (4.4)	8503 (4.6)	5646 (4.1)	885 (4.4)	1251 (3.0)	3303 (6.0)	1358 (7.9)	854 (2.5)	≤.001
38‐40 + 6	367 792 (74.8)	149 685 (80.5)	92 235 (67.5)	15 646 (78.8)	28 202 (67.7)	49 328 (88.9)	13 016 (75.6)	19 680 (56.5)	
41‐41 + 6	101 998 (20.7)	27 695 (14.9)	38 770 (28.4)	3334 (16.8)	12 178 (29.3)	2868 (5.1)	2842 (16.5)	14 311 (41.1)	
Birthweight									
≤2500 g	4993 (1.0)	1618 (0.8)	1496 (1.1)	174 (0.9)	362 (0.9)	729 (1.3)	218 (1.3)	396 (1.1)	≤.001
2500‐3499 g	240 896 (49.0)	97 316 (52.4)	63 789 (46.6)	10 629 (53.5)	19 811 (47.6)	28 020 (50.5)	8195 (47.6)	13 136 (37.7)	
3500‐3999 g	176 014 (35.8)	64 872 (34.9)	50 361 (36.9)	6947 (35.0)	15 558 (37.4)	19 029 (34.3)	5692 (34.6)	13 285 (38.1)	
≥4000 g	69 687 (14.2)	22 077 (11.9)	21 005 (15.4)	2115 (10.6)	5900 (14.2)	7721 (13.9)	2841 (16.5)	8028 (23.0)	
Small‐for‐gestational age	2151 (0.4)	720 (0.4)	594 (0.4)	88 (0.4)	170 (0.4)	327 (0.6)	177 (0.7)	135 (0.4)	≤.001
Large‐for‐gestational age	6182 (1.3)	1165 (0.6)	1990 (1.5)	151 (0.8)	626 (1.5)	871 (1.6)	324 (1.9)	994 (2.9)	≤.001
Birth trauma child^d^	16 460 (3.3)	2954 (1.6)	2530 (1.9)	2570 (12.9)	5475 (13.2)	765 (1.4)	689 (4.0)	1477 (4.2)	≤.001

a Induction or augmentation with oxytocin, prostaglandin, and/or artificial rupture of membranes.

b Socioeconomic status are index data of relative socioeconomic advantage and disadvantage, low (deciles 0‐3), middle (deciles 4‐6), high (7‐10 deciles).

Pharmacological pain medication (ie, nitrous oxide, systematic opioid, local administered to perineum, pudendal, morphine, and pethidine).

Birth trauma refers to birth trauma to central or peripheral nervous system, birth trauma to scalp, birth trauma to skeleton, intracranial laceration, and hemorrhage due to birth trauma.

### 3.1 Outcomes at short‐term follow‐up (first 28 days)

The prevalence of jaundice, feeding problems, and hypothermia were, respectively, 4%, 3%, and 2%. Compared with infants who were born by spontaneous vaginal birth, all other infants born with either medical or operative birth interventions had significantly higher odds of jaundice and feeding problems, except for an elective cesarean delivery which was not associated with the risk of jaundice (*P* = .07). Infants born by instrumental vaginal birth after induction or augmentation showed the highest association of jaundice (crude OR 3.26 [95% CI 3.12‐3.41], adjusted OR [aOR] 2.75 [95% CI 2.61‐2.91]). Significantly higher odds of hypothermia were observed for infants born by all the specified cesarean groups compared with those born by spontaneous vaginal delivery (Table 2).

**Table 2:** Prevalence and associations between birth interventions and short‐term child health outcomes, New South Wales, Australia, 2000‐2013

Short‐term adverse health outcomes	Total population		
	No. of events N (%)	Unadjusted OR (95% CI)	Adjusted^a^ OR (95% CI)
Jaundice			
Spontaneous vaginal birth	5299 (2.9)	Reference	Reference
Vaginal birth with induction or augmentation	4986 (3.6)	**1.28 (1.23‐1.33)** b	**1.36 (1.31‐1.42)**
Instrumental vaginal birth without induction or augmentation	1615 (8.1)	**3.01 (2.84‐3.19)**	**2.34 (2.20‐2.49)**
Instrumental vaginal birth with induction or augmentation	3662 (8.8)	**3.26 (3.12‐3.41)**	**2.75 (2.61‐2.91)**
Elective cesarean	1638 (3.0)	1.02 (0.96‐1.07)	1.07 (1.00‐1.14)
Emergency cesarean without induction or augmentation	686 (4.0)	**1.39 (1.29‐1.51)**	**1.24 (1.14‐1.36)**
Emergency cesarean after induction or augmentation	1375 (3.9)	**1.38 (1.30‐1.46)**	**1.31 (1.22‐1.41)**
Feeding problems			
Spontaneous vaginal birth	1886 (1.0)	Reference	Reference
Vaginal birth with induction or augmentation	1907 (1.4)	**1.37 (1.28‐1.46)**	**1.23 (1.15‐1.32)**
Instrumental vaginal birth without induction or augmentation	513 (2.6)	**2.58 (2.34‐2.85)**	**1.44 (1.30‐1.60)**
Instrumental vaginal birth with induction or augmentation	1344 (3.2)	**3.22 (3.00‐3.46)**	**1.73 (1.59‐1.89)**
Elective cesarean	1095 (2.0)	**1.93 (1.79‐2.08)**	**1.81 (1.64‐1.99)**
Emergency cesarean without induction or augmentation	450 (2.6)	**2.58 (2.33‐2.87)**	**1.82 (1.61‐2.05)**
Emergency cesarean after induction or augmentation	1090 (3.1)	**3.10 (2.87‐3.34)**	**1.85 (1.67‐2.04)**
Hypothermia			
Spontaneous vaginal birth	5537 (3.0)	Reference	Reference
Vaginal birth with induction or augmentation	4484 (3.3)	**1.09 (1.05‐1.14)**	1.04 (1.00‐1.08)
Instrumental vaginal birth without induction or augmentation	687 (3.5)	**1.16 (1.07‐1.26)**	0.96 (0.88‐1.04)
Instrumental vaginal birth with induction or augmentation	1542 (3.7)	**1.24 (1.17‐1.31)**	1.01 (0.94‐1.08)
Elective cesarean	2104 (3.8)	**1.26 (1.20‐1.32)**	**1.16 (1.08‐1.24)**
Emergency cesarean without induction or augmentation	742 (4.3)	**1.45 (1.34‐1.56)**	**1.24 (1.13‐1.36)**
Emergency cesarean after induction or augmentation	1775 (5.1)	**1.72 (1.63‐1.82)**	**1.43 (1.33‐1.54)**

a Adjusted for maternal characteristics (ie, maternal age, country of birth, socioeconomic status, parity), birth characteristics (ie, pharmacological pain medication or anesthesia), and child characteristics (ie, gender, gestational age, birthweight, small‐for‐gestational age, large‐for‐gestational age, birth trauma).

b Associations reported in bold reflect a statistical significant association (*P* ≤ .01).

### 3.2 Outcomes at longer‐term follow‐up (up to 5 years of age)

Diagnosed respiratory infections had the highest prevalence of any of the medical conditions during the 5‐year follow‐up period, 14%. The lowest prevalence was observed for gastrointestinal disorders, 0.5%. Other bacterial infections, sepsis, otitis, cystitis, or urethritis, were reported in 8% of the children in the study. There was no evidence to suggest an association between mode of birth and the odds of asthma. Metabolic disorder was reported in 1% of children and 3% were diagnosed with eczema. Compared with children born after spontaneous vaginal birth without induction or augmentation, all other groups had higher odds of respiratory infections, metabolic disorder, and eczema (Table 3). Odds of gastrointestinal disorders were higher among children born after vaginal birth with induction or augmentation and after elective cesarean delivery. Other infections were more prevalent among all exposure groups compared with those born after spontaneous vaginal birth without induction or augmentation. No statistical significant associations between other infections and groups born after instrumental birth either without or with induction or augmentation were observed (*P*‐values .07 and .02, respectively). Compared with children born by spontaneous vaginal birth, children born by cesarean delivery had higher odds of longer‐term adverse health outcomes. Birth by elective cesarean delivery aOR 2.49 (95% CI 2.19‐2.82), an emergency cesarean aOR 2.63 (95% CI 2.26‐3.07), and emergency cesarean delivery after induction aOR 2.41 (95% CI 2.11‐2.76) was associated with increased odds of metabolic disorder.

**Table 3:** Prevalence and associations between birth interventions and longer‐term child health outcomes, New South Wales, Australia, 2000‐2013

Longer‐term adverse health outcomes	Total population		
	No. of events N (%)	Unadjusted OR (95% CI)	Adjusted^a^ OR (95% CI)
Asthma			
Spontaneous vaginal birth	5738 (3.1)	Reference	Reference
Vaginal birth with induction or augmentation	4294 (3.1)	1.01 (0.97‐1.05)	1.01 (0.96‐1.05)
Instrumental vaginal birth without induction or augmentation	640 (3.2)	1.04 (0.96‐1.13)	1.07 (0.98‐1.17)
Instrumental vaginal birth with induction or augmentation	1201 (2.9)	0.92 (0.87‐0.98)	0.97 (090‐1.04)
Elective cesarean	1868 (3.4)	1.07 (1.02‐1.13)	1.04 (0.97‐1.11)
Emergency cesarean without induction or augmentation	604 (3.5)	**1.12 (1.03‐1.23)** b	1.09 (0.99‐1.20)
Emergency cesarean after induction or augmentation	1084 (3.1)	0.99 (0.93‐1.06)	1.03 (0.95‐1.12)
Respiratory infections			
Spontaneous vaginal birth	22 454 (12.1)	Reference	Reference
Vaginal birth with induction or augmentation	18 653 (13.7)	**1.14 (1.12‐1.16)**	**1.11 (1.08‐1.13)**
Instrumental vaginal birth without induction or augmentation	2960 (14.9)	**1.27 (1.22‐1.33)**	**1.25 (1.20‐1.31)**
Instrumental vaginal birth with induction or augmentation	6538 (15.7)	**1.34 (1.30‐1.38)**	**1.31 (1.27‐1.36)**
Elective cesarean	9660 (17.4)	**1.50 (1.46‐1.54)**	**1.35 (1.31‐1.40)**
Emergency cesarean without induction or augmentation	3030 (17.6)	**1.53 (1.47‐1.60)**	**1.39 (1.32‐1.46)**
Emergency cesarean after induction or augmentation	5497 (15.8)	**1.34 (1.30‐1.38)**	**1.29 (1.23‐1.34)**
Gastrointestinal disorders			
Spontaneous vaginal birth	665 (0.4)	Reference	Reference
Vaginal birth with induction or augmentation	665 (0.5)	**1.35 (1.21‐1.50)**	**1.22 (1.09‐1.37)**
Instrumental vaginal birth without induction or augmentation	97 (0.5)	**1.36 (1.10‐1.69)**	1.13 (0.90‐1.41)
Instrumental vaginal birth with induction or augmentation	181 (0.4)	1.20 (1.02‐1.42)	0.96 (0.79‐1.16)
Elective cesarean	330 (0.6)	**1.63 (1.43‐1.86)**	1.21 (1.02‐1.44**)**
Emergency cesarean without induction or augmentation	101 (0.6)	**1.62 (1.31‐2.00)**	1.24 (0.98‐1.57)
Emergency cesarean after induction or augmentation	193 (0.6)	**1.53 (1.30‐1.79)**	1.19 (0.98‐1.45)
Other infections			
Spontaneous vaginal birth	13 448 (7.2)	Reference	Reference
Vaginal birth with induction or augmentation	11 750 (8.6)	**1.20 (1.16‐1.23)**	**1.12 (1.09‐1.15)**
Instrumental vaginal birth without induction or augmentation	1678 (8.4)	**1.18 (1.12‐1.24)**	1.05 (1.00‐1.11)
Instrumental vaginal birth with induction or augmentation	3589 (8.6)	**1.20 (1.15‐1.24)**	1.06 (1.01‐1.10)
Elective cesarean	5326 (9.6)	**1.33 (1.29‐1.38)**	**1.07 (1.03‐1.12)**
Emergency cesarean without induction or augmentation	1630 (9.5)	**1.32 (1.25‐1.39)**	**1.10 (1.04‐1.17)**
Emergency cesarean after induction or augmentation	3218 (9.2)	**1.28 (1.23‐1.33)**	**1.10 (1.05‐1.16)**
Metabolic disorder			
Spontaneous vaginal birth	1041 (0.6)	Reference	Reference
Vaginal birth with induction or augmentation	1124 (0.8)	**1.46 (1.34‐1.59)**	**1.35 (1.23‐1.48)**
Instrumental vaginal birth without induction or augmentation	181 (0.9)	**1.63 (1.39‐1.91)**	**1.28 (1.08‐1.52)**
Instrumental vaginal birth with induction or augmentation	463 (1.1)	**1.98 (1.77‐2.21)**	**1.54 (1.35‐1.75)**
Elective cesarean	919 (1.7)	**2.93 (2.68‐3.21)**	**2.49 (2.19‐2.82)**
Emergency cesarean without induction or augmentation	338 (2.0)	**3.51 (3.10‐3.97)**	**2.63 (2.26‐3.07)**
Emergency cesarean after induction or augmentation	653 (1.9)	**3.34 (3.02‐3.68)**	**2.41 (2.11‐2.76)**
Eczema			
Spontaneous vaginal birth	3566 (1.9)	Reference	Reference
Vaginal birth with induction or augmentation	3529 (2.6)	**1.34 (1.28‐1.41)**	**1.16 (1.10‐1.22)**
Instrumental vaginal birth without induction or augmentation	1171 (5.9)	**3.20 (2.99‐3.42)**	**2.18 (2.03‐2.35)**
Instrumental vaginal birth with induction or augmentation	2817 (6.8)	**3.68 (3.50‐3.87)**	**2.30 (2.16‐2.45)**
Elective cesarean	1541 (2.8)	**1.43 (1.35‐1.52)**	**1.11 (1.03‐1.19)**
Emergency cesarean without induction or augmentation	1178 (6.8)	**3.71 (3.46‐3.97)**	**2.54 (2.35‐2.75)**
Emergency cesarean after induction or augmentation	2476 (7.1)	**3.85 (3.65‐4.06)**	**2.38 (2.22‐2.55)**

a Adjusted for maternal characteristics (ie, maternal age, country of birth, socioeconomic status, parity), birth characteristics (ie, pharmacological pain medication or anesthesia), and child characteristics (ie, gender, gestational age, birthweight, small‐for‐gestational age, large‐for‐gestational age, birth trauma).

b Associations reported in bold reflect a statistical significant association (*P* ≤ .01).

## 4 DISCUSSION

The aim of this study was to examine the association between medical birth interventions and/or operative birth interventions on short‐ and longer‐term child health outcomes in healthy women and their children by analyzing population‐based linked data. Our results showed that newborns born by instrumental birth after induction or augmentation were more likely to experience jaundice. Children born by cesarean delivery were particularly at increased risk for adverse health outcomes in the longer term, that is, respiratory infection, other infection, and metabolic disorder.

There is emerging evidence that some birth interventions may have an effect on the neonatal immune response and the child's health in the longer term.^7^, ^29^ There is evidence of short‐term health impacts for the infant after a cesarean delivery, such as hypothermia, impaired lung function, altered metabolism, altered blood pressure, and altered feeding, which is consistent with our results.^7^, ^27^, ^28^ Some of these changes might be due to a lack of labor stress, associated with physiological maladaptation after birth. Some epidemiological studies have linked the mode of birth (particularly cesarean delivery) to increasing rates of asthma and gastrointestinal disorders.^15^, ^17^ However, other epidemiological studies did not report higher rates of asthma, diabetes type 1, obesity, and inflammatory bowel disease for children born with birth interventions.^12^, ^16^ Several studies that included meta‐analyses reported that children born by cesarean delivery were at higher risk of developing obesity, diabetes, or asthma in childhood.^4^, ^6^, ^30^, ^31^

The EPIIC hypothesis postulated by some of the authors in this paper, proposes that nonphysiological interventions during the intrapartum period, and specifically the use of synthetic oxytocin, epidural analgesia, and cesarean delivery, may interrupt the normal stress of being born.^10^, ^11^ This could have an epigenetic effect on specific genes, such as those that program immune responses, including weight regulation and metabolism. In support of an epigenetic hypothesis in this area, an association between mode of birth and DNA methylation has previously been reported.^32^, ^33^ Schlinzig et al examined 37 term babies born by elective cesarean delivery (n* *=* *16) or vaginal birth (n* *=* *21) and found a higher global measure of DNA methylation if the infant was born by cesarean delivery. While there was a nonsignificant difference between vaginal birth and cesarean delivery at 3‐5 days postpartum, the pattern did not alter in the infants born vaginally but significantly decreased in infants born by cesarean delivery.^33^ Almgren et al^32^ undertook a more precise analysis, looking at DNA from hematopoietic stem cells (CD34+). Those in specific gene sites that programmed for immune‐mediated disease showed different methylation patterns in infants born by cesarean delivery than those born vaginally.^32^ Furthermore, those infants born after shorter labor showed similar DNA methylation patterns to those born by cesarean, suggesting that physiological labor stress over a certain period of time is required to program certain autoimmune responses in the neonate.

An alternative theory, the Extended Hygiene Hypothesis suggests that in utero, during a vaginal birth, and following skin‐to‐skin contact and breastfeeding, the infant needs to gather a community of microbes that come from the mother and the surrounding environment.^8^, ^9^ Establishing the gut microbiota may be important in protecting the child, and later the adult, against atopic and immunological diseases.^10^, ^34^ Disturbances in this process could be linked to developing infectious, inflammatory, and allergic diseases later in life.^10^, ^34^ However, some studies associate the mode of birth with differences in the child's microbiota, other conflicting results showed that there was no effect of cesarean delivery on the early microbiota beyond the immediate neonatal period.^35^

Despite this, there is a global awareness that cesarean delivery rates are too high. Currently, the emphasis is on labor induction to address this issue, as in, for example, a recently reported randomized controlled trial on routine induction of labor at 39 weeks in nulliparous women.^36^ Although this study showed a reduced cesarean delivery, our population level analysis shows that replacing one technical intervention with another might not improve longer‐term outcomes. We suggest that those looking to reduce unnecessary intervention could consider results of systematic reviews that show that relationship‐based interventions, such as continuous support in labor, or continuity of midwifery care, are associated with decreased interventions, improved rates of physiological birth, and higher levels of maternal reports of well being, without adversely affecting mortality and morbidity, and at reduced cost for women and health systems.^37^, ^38^

Our study had several strengths and limitations. To our knowledge, this is the first study that has provided an overview of associations between all possible birth interventions and a wide range of adverse child health outcomes within a large population of healthy pregnant women and their children. In our analyses, we adjusted for a range of confounders, including maternal characteristics, birth characteristics, and child characteristics. However, our associations could still be affected by unmeasured confounding, such as maternal body mass index, antibiotic use during pregnancy or administered during childhood until the age of 5, breast or artificial feeding, paternal characteristics, and familial environmental and genetic factors. Moreover, we were unable to control for confounding by indication since the underlying reasons for the provided medical and operative birth interventions were unknown.^39^ All of these factors may independently be associated with some of the health outcomes seen in children and therefore our findings must be interpreted with caution. It is possible that the routine use of intrapartum antibiotics also plays a role in the disturbance of the microbiome. As a consequence, the infant may experience adverse outcomes. Furthermore, experimental and laboratory‐based studies are needed to determine the precise mechanism and contribution of the different factors to the outcome. While we included country of birth, we could not include ethnicity and this may also affect outcomes and associations. We were only able to examine admissions of the child to a hospital while visits to general practitioners were not incorporated, suggesting an underreporting of adverse outcomes. Unfortunately, population‐based linked data are restricted to the selection of variables and limited ability to verify the accuracy of the data, but do provide a cost‐effective way of establishing incidence and association of (rare) health outcomes and can direct future research.

Further research is required to confirm or refute the findings from this study. Research ideally would include other population‐based data registries, including a longer follow‐up period for a wider range of adverse child health outcomes, particularly those that are found more commonly beyond 5 years of age (eg, asthma). More research is also needed to explain some of the potential mechanisms at play, including epigenetic and microbiome research.

By analyzing linked population data, we obtained insight into the association of medical and operative birth interventions and short‐ and longer‐term child health outcomes. These results support the “Too little too late, too much too soon debate” in maternal care, in which Miller et al^40^ argued that unnecessary use of nonevidence‐based interventions can be harmful for healthy women and infants, as much as a lack of lifesaving interventions is damaging for those that need them. Our results should make consumers and maternal health care professionals aware of the potential harm that birth interventions may have in the longer term, encouraging a “precautionary principle” approach that weighs the possible benefits of the intervention against its potential detrimental effects for each mother and child.^41^ The aim should always be to provide the right amount of care at the right time in the right way to childbearing women, with a clear assessment of the potential consequences of just‐in‐case interventions.^40^

## 5 CONCLUSION

Children born by spontaneous vaginal birth had fewer short‐ and longer‐term health problems, compared with those born after birth interventions. This suggests that when examining labor interventions, researchers need to pay attention to use of exogenous oxytocin and to instrumental and operative birth, and that follow‐up should be continued into the longer term.
