# Supplementary material for: The effect of medical and operative birth interventions on child health outcomes in the first 28 days and up to 5 years of age: A linked data population‐based cohort study
Source: Birth. 2018 Mar 25;45(4):347–57. doi: 10.1111/birt.12348 (PMC6282837; doi:10.1111/birt.12348)
Supplement: Supplementary file 1 [file BIRT-45-347-s001.docx]

**Supporting Information**

**Overview of the analysed International Statistical Classification of Diseases and Related Health Problems, Tenth Revision, Australian Modification (ICD-10-AM) codes**

|  | | **ICD- Coding specified** | **ICD-AM Codes** |
| --- | --- | --- | --- |
|  | |  |  |
| **CHILD CHARACTERISTICS** | |  |  |
|  | **Birth trauma** | Intracranial laceration and haemorrhage due to birth trauma | P10 |
|  |  | Other birth trauma to central nervous system | P11 |
|  |  | Birth trauma to scalp | P12 |
|  |  | Birth trauma to skeleton | P13 |
|  |  | Birth trauma to peripheral nervous system | P14 |
|  |  | Other birth trauma | P15 |
|  | **Small for gestational age** | Disorders related to short gestation and low birth weight | P07 |
|  | **Large for gestational age** | Disorders related to long gestation and high birth weight | P08 |
|  |  |  |  |
| **SHORT TERM HEALTH OUTCOMES** | | |  |
|  | **Jaundice** | Jaundice due to isoimmunisation | P55-P57 |
|  |  | Neonatal jaundice due to other excessive haemolysis | P58 |
|  |  | Neonatal jaundice from other unspecified causes | P59 |
|  | **Feeding problems** | Feeding problems of the newborn | P92 |
|  | **Hypothermia** | Hypothermia of newborn | P80 |
|  |  | Other disturbances of temperature regulation of newborn | P81 |
|  |  | Other conditions of integument specific to foetus and newborn | P83 |
|  |  |  |  |
| **LONG TERM HEALTH OUTCOMES** | | |  |
|  | **Asthma** | Asthma and status asthmaticus | J45 |
|  |  | Status asthmaticus | J46 |
|  | **Respiratory infections** | Birth asphyxia | P21 |
|  |  | Respiratory distress of newborn | P22 |
|  |  | Neonatal aspiration syndrome | P24 |
|  |  | Air leak syndrome originating in the perinatal period | P25 |
|  |  | Pulmonary haemorrhage originating in the perinatal period | P26 |
|  |  | Chronic respiratory disease originating in the perinatal period | P27 |
|  |  | Other respiratory conditions originating in the perinatal period | P28 |
|  |  | Common cold | J00 |
|  |  | Acute pharyngitis | J02 |
|  |  | Acute tonsillitis | J03 |
|  |  | Acute laryngitis and trachitis | J04 |
|  |  | Acute obstructive laryngitis | J05 |
|  |  | Acute upper respiratory infections of multiple and unspecified sites | J06 |
|  |  | Influenza due to certain identified influenza virus | J09 |
|  |  | Influenza due to other identified influenza virus | J10 |
|  |  | Influenza, virus not specified | J11 |
|  |  | Viral pneumonia, not elsewhere specified | J12 |
|  |  | Pneumonia due to Streptococcus pneumonia | J13 |
|  |  | Pneumonia due to Haemophilus influenza | J14 |
|  |  | Bacterial pneumonia not elsewhere specified | J15 |
|  |  | Pneumonia due to other infectious organism not elsewhere specified | J16 |
|  |  | Pneumonia in diseases classified elsewhere | J17 |
|  |  | Acute bronchitis | J20-J21 |
|  |  | Unspecified acute lower respiratory infection | J22 |
|  |  | Chronic rhinitis, nasopharyngitis, and pharyngitis | J31 |
|  | **Gastrointestinal disorders** | Necrotizing enterocolitis of newborn | P77 |
|  |  | Other perinatal digestive system disorder | P78 |
|  |  | Gastritis and duodenitis | K29 |
|  | **Other infections** | Bacterial sepsis of newborn | P36 |
|  |  | Other infections specific to the perinatal period | P39 |
|  |  | Streptococcal Sepsis | A40 |
|  |  | Other sepsis | A41 |
|  |  | Streptococcus and staphylococcus as the cause of disease classified to other chapters | B95 |
|  |  | Other bacterial agents as the cause of diseases classified to other chapters | B96 |
|  |  | Viral agents as the cause of diseases classified to other chapters | B97 |
|  |  | Otitis externa | H60 |
|  |  | Non-supportive otitis media | H65 |
|  |  | Suppurative and unspecified otitis media | H66 |
|  |  | Otitis media in diseases classified elsewhere | H67 |
|  |  | Mastoiditis and related condition | H70 |
|  |  | Cystitis | N30 |
|  |  | Urethritis and urethral syndrome | N34 |
|  | **Metabolic disorder** | Transitory disorders of carbohydrate metabolism specific for fetus/newborn | P70 |
|  |  | Intermediate hyperglycaemia | E09 |
|  |  | Diabetes Mellitus type 1 | E10 |
|  |  | Diabetes Mellitus type 2 | E11 |
|  |  | Other specified diabetes mellitus | E13 |
|  |  | Localised adiposity | E65 |
|  |  | Hypocalcaemia | E66 |
|  |  | Other hyper alimentation | E67 |
|  | **Eczema** | Atopic dermatitis | L20 |
|  |  | Seborrheic dermatitis | L21 |
|  |  | Diaper (napkin) dermatitis | L22 |
|  |  | Allergic contact dermatitis | L23 |
|  |  | Irritant contact dermatitis | L24 |
|  |  | Unspecified contact dermatitis | L25 |
|  |  | Exfoliative dermatitis | L26 |
|  |  | Dermatitis due to substances taken internally | L27 |
|  |  | Lichen Simplex chronicus and prurigo | L28 |
|  |  | Pruritus | L29 |
|  |  | Other dermatitis | L30 |
